# Prognostic value of whole-body diffusion-weighted imaging with background body signal suppression in CRPC patients undergoing Ra-223 therapy: an exploratory analysis

**DOI:** 10.1007/s11604-025-01817-2

**Published:** 2025-06-17

**Authors:** Yumiko Kono, Keita Utsunomiya, Satoaki Nakamura, Yasuhiro Ueno, Kaoru Maruyama, Junichi Ikeda, Kenta Takayasu, Nae Takizawa, Hisanori Taniguchi, Masaaki Yanishi, Hidefumi Kinoshita, Noboru Tanigawa

**Affiliations:** 1 Department of Radiology, Kansai Medical University, 2-5-1 Shin-machi, Hirakata City, Osaka 573-1010 Japan; 2 Department of Urology and Andrology, Kansai Medical University, 2-5-1 Shin-machi, Hirakata City, Osaka 573-1010 Japan

**Keywords:** Prostatic neoplasms, Castration-resistant, Castration-resistant prostate cancer, Radium-223, Bone neoplasms, Diffusion magnetic resonance imaging

## Abstract

**Objectives:**

The aim of this study was to evaluate the utility of diffusion-weighted whole-body imaging with background body signal suppression (DWIBS) in monitoring the response to Ra-223 therapy in patients with castration-resistant prostate cancer (CRPC) and bone metastasis.

**Materials and methods:**

This retrospective study included 15 patients with CRPC and bone metastases. DWIBS scans were performed at baseline and after three cycles of Ra-223 therapy. Quantitative analysis of tumor total diffusion volume (tDV) categorized patients as stable disease (DWIBS-SD), partial response (DWIBS-PR), or progressive disease (DWIBS-PD). Kaplan–Meier analysis and log-rank tests were used to assess the correlation between DWIBS findings and survival.

**Results:**

Of the 15 patients (median age 72 years ± 7.3), 7 (47%) were classified as DWIBS-SD, 3 (20%) as DWIBS-PR, and 5 (33%) as DWIBS-PD. DWIBS-PD group had significantly shorter survival than the DWIBS-non-PD group (*P* = 0.004). Despite no significant differences in age, alkaline phosphatase, prostate-specific antigen or bone metastasis volume, DWIBS-PD group had a significantly higher proportion of patients with Eastern Cooperative Oncology Group performance status score of 2 before the treatment compared to DWIBS-non-PD group (*P* = 0.039).

**Conclusion:**

DWIBS is a valuable tool for monitoring treatment response and predicting outcome in patients with CRPC undergoing Ra-223 therapy. Early intervention or treatment modification is recommended for patients with DWIBS-PD.

**Supplementary Information:**

The online version contains supplementary material available at 10.1007/s11604-025-01817-2.

## Introduction

Castration-resistant prostate cancer (CRPC) is a prostate cancer characterized by continued disease progression, despite androgen deprivation therapy. Bone metastases are prevalent and serious complications of CRPC, which contribute to increased morbidity and diminished quality of life [1, 2]. Radium-223 (Ra-223), an alpha-emitting radiopharmaceutical, is introduced as a bone-targeting therapeutic agent. Ra-223 accumulates in the areas of increased bone turnover, thereby mitigating skeletal-related events and enhancing the overall survival [3, 4]. Despite its efficacy, the antitumor effects of Ra-223 are confined to bone metastases and do not extend to soft tissue or visceral metastases. Although the clinical benefits of Ra-223 have been well documented to adequately assess therapeutic efficacy of Ra-223, comprehensive evaluation of bone and other organ systems is required. Biomarkers, such as prostate-specific antigen (PSA), alongside with conventional imaging modalities, are traditionally employed for patient stratification and response monitoring but have demonstrated limited utility in assessing Ra-223 therapy [5]. Moreover, established imaging techniques, such as bone scintigraphy, CT, and MRI, have inherent limitations in comprehensive whole-body disease assessment in advanced cancer cases [6].

Diffusion-weighted whole-body imaging with background body signal suppression (DWIBS) is an emerging imaging modality that facilitates comprehensive, noninvasive whole-body evaluation without the need of contrast agents [7, 8]. DWIBS offers valuable functional insights into tissue cellularity and integrity, making it a promising technique for detecting, characterizing, and monitoring metastatic lesions [9]. The ability to simultaneously visualize both bone and soft tissue metastases has a significant advantage in evaluating CRPC, because metastatic spread is often multifocal. In addition, DWIBS enables quantitative assessment of metastatic bone lesions by calculating the tumor total diffusion volume (tDV), which is a comprehensive indicator of metastatic burden and response to therapy. Clinical trials such as the ALSYMPCA trial [10] have established Ra-223 as a viable therapeutic option, but questions remain concerning the ideal timing of intervention and the identification of patient subgroups most likely to benefit from Ra-223 therapy [3, 11]. To our knowledge, clinical application of DWIBS during Ra-223 therapy in CRPC has not been fully explored. Accordingly, this study aimed to assess the utility of DWIBS in CRPC patients undergoing Ra-223 therapy and sought to determine whether DWIBS can effectively identify therapeutic effects of Ra-223 therapy and patient prognosis in a clinical setting.

## Materials and methods

This study was approved by the Kansai Medical University Ethics Committee (approval number 2022180) and conducted in accordance with the Declaration of Helsinki.

### Patients

This study included 15 patients with CRPC and bone metastases who were diagnosed at Kansai Medical University and initiated Ra-223 therapy between March 1, 2019, and December 31, 2023. DWIBS scans were performed at 2 time points: baseline (before the start of Ra-223 therapy) and after the 3rd cycle of Ra-223 therapy. The eligibility criteria were male patients aged 18 years and older with a confirmed diagnosis of CRPC based on clinical, laboratory and imaging criteria. All patients had bone metastases identified by imaging (CT, MRI and/or bone scintigraphy) and had survival data for at least 1 year after the start of treatment. Exclusion criteria were: patients with incomplete or missing DWIBS image data at baseline and at the 3-month follow-up (1 patient), patients with no followed-up data and survival data at one year after the start of the treatment (2 patients), and patients who died of causes other than cancer during the follow-up period (1 patient).

### Radium-223 therapy protocol

Ra-223 was administered intravenously at a dose of 55 kBq/kg every 4 weeks for 6 cycles according to the standard clinical protocol. Comprehensive care, including symptom management of bone-related events and regular clinical and imaging assessments, was provided to all the patients.

### DWIBS imaging

DWIBS scans were obtained at two specified time points, and all scans were performed using a 1.5-T system (Achieva 1.5 T dStream; Philips) with standardized imaging parameters to ensure consistency (Table 1). The scans were acquired in axial sections from the skull base to the pelvis focusing bone metastases and soft tissue invasion. All DWIBS images were evaluated by a single board-certified radiologist (with 14 years of experience in diagnostic radiology). Quantitative analysis was performed to calculate the total volume of bone metastases, referred to as tDV. The tDV quantifies the total extent of bone metastases with restricted diffusion, as identified by apparent diffusion coefficient values below a specified threshold, thereby serving as an objective measure of the metastatic disease burden. Patients were classified into three categories based on the change in tDV: partial response (DWIBS-PR) was defined as a decrease in the volume of bone metastases by ≥ 10 mL and ≥ 10% (relative to baseline) with no new lesions; progressive disease (DWIBS-PD) was defined as an increase in the volume of bone metastases by ≥ 10 mL and ≥ 10% (relative to baseline), or the appearance of multiple new bone lesions or new organ metastases; and stable disease (DWIBS-SD) was defined as not meeting DWIBS-PR or DWIBS-PD criteria and showing no significant worsening or regression of bone metastases.

**Table 1:** MRI sequence parameters

Image parameters	T2WI	DWIBS	mDIXON
Sequence type	TSE	EPI	FFE
TSE/EPI factor	57	41	–
TR/TE/Tl (ms)	10,000/90/–	4500/75/183	6.7/2/-
Slice thickness/gap (mm)	6/−1	6/−1	5.5/0
FOV (mm)	450	450	450
Acquired voxel size (mm^3^)	1.29/1.61/6	3.5/3.54/6	0.8/1.2/5.5
Reconstructed voxel size (mm^3^)	0.88/0.88/6	1.56/1.56/6	0.78/0.78/5.5
Slice thickness (mm)	6	6	5.5
B-value(mm^2^/s)	–	0 and 1000	–

*T2WI* T2-weighted imaging, *DWIBS* diffusion-weighted imaging with background body signal suppression, *mDIXON* modified DIXON, *TSE* turbo spine echo, *EPI* echo-planar imaging, *TR* repetition time, *TE* echo time, *Tl* time inversion, *FOV* field of view

### Response classification

Based on changes in tDV and the presence or absence of new lesions, patients were classified into DWIBS-PD or DWIBS-non-PD. Specifically, DWIBS-PD was defined as an increase in tDV of ≥ 10 mL and ≥ 10% (relative to baseline) or the appearance of new lesions. Patients who did not meet the DWIBS-PD criteria were collectively designated as DWIBS-non-PD, which included both DWIBS-PR and DWIBS-SD.

### Quantitative analysis

**Fig. 1:**
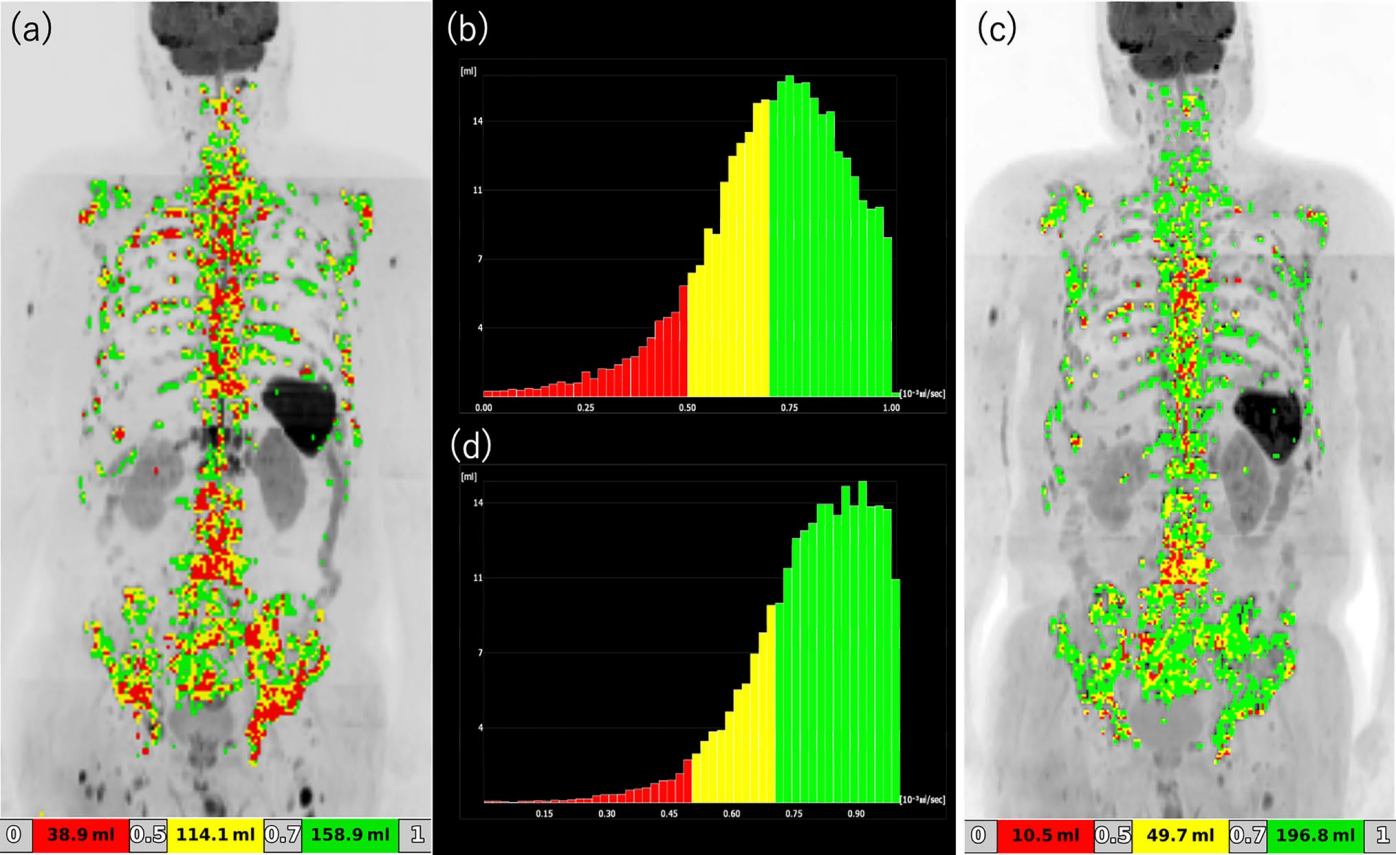
DWIBS-partial response (DWIBS-PR) case—baseline vs. post-cycle-3. **a** Baseline DWIBS maximum-intensity-projection (MIP) image with ADC color overlay. **b** Baseline ADC histogram of segmented bone metastases. **c** Post-cycle-3 DWIBS MIP with ADC overlay. **d** Corresponding post-cycle-3 ADC histogram. Red indicates ADC < 0.5 × 10⁻^3^ mm^2^/s, yellow 0.5–0.7 × 10⁻^3^ mm^2^/s, and green 0.7–1.0 × 10⁻^3^ mm^2^/s. The tumor total diffusion volume (voxels with ADC values ≤ 1.0 × 10⁻^3^ mm^2^/s) decreased from 345.4 mL to 257.0 mL, indicating a partial response

Using BD Score software (ver. 1.4.003; PixSpace, Tokyo, Japan), a semi-automated platform designed to extract diffusion-restricted bone lesions based on ADC thresholds, we extracted voxels in the bone that had an ADC value of ≤ 1.0 × 10^−3^ mm^2^/s. The ADC color map was generated to highlight 3 subthresholds: red for < 0.5, yellow for 0.5–0.7, and green for 0.7–1.0 × 10^−3^ mm^2^/s (Fig. 1). High-signal normal structures (e.g., spleen, spinal cord, intestinal contents, and testes), and extra-osseous signals were manually removed. Additionally, lesions in the skull and limb bones—which were imaged differently depending on the patient—underwent manual editing to ensure accurate quantification. For unclear bone lesions, T2WI and mDIXON images, as well as pretreatment bone scintigraphy, were reviewed to determine whether they were metastatic or non-metastatic. All editing was carried out by 1 certified radiologist.

### Patient survival

The primary endpoint of this study is overall survival during the first year after the start of Ra-223 therapy. Patients were divided into 2 groups: those who survived for 1 year after the start of the treatment and those who died within 1 year. Age, Eastern Cooperative Oncology Group (ECOG) Performance Status (PS), which is a widely used indicator of patients’ functional status, bone pain, body mass index (BMI), number of Ra-223 treatments, Gleason score, alkaline phosphatase (ALP), PSA levels, PSA doubling time, bone scintigraphy index (BSI), tDV before treatment and DWIBS assessments during treatment were investigated to determine the differences between the two groups.

### Statistical analysis

The Wilcoxon rank-sum test was applied for continuous variables and the Pearson chi-square test and Fisher’s exact test were used for binary variables. The treatment effect of DWIBS was evaluated using Kaplan–Meier survival analysis and log-rank test. Statistical significance was set at *P* < 0.05.

## Results

### Patient characteristics

**Fig. 2:**
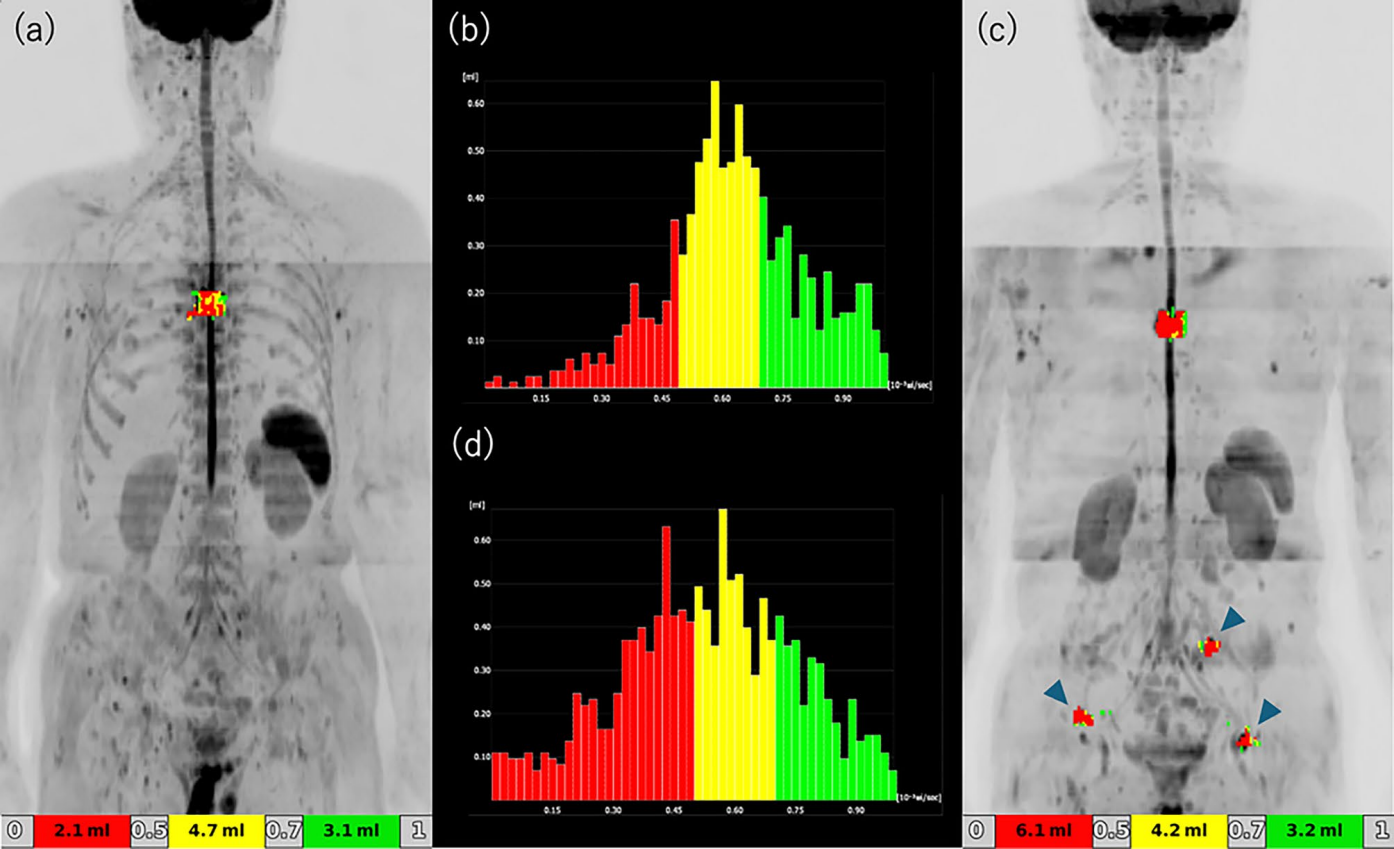
DWIBS-progressive disease (DWIBS-PD) case 1—bone-dominant progression. **a** Baseline DWIBS MIP with ADC colour overlay. **b** Baseline ADC histogram. **c** Follow-up DWIBS MIP after cycle 3 showing new bone lesions (arrowheads). **d** Follow-up ADC histogram. The tDV increased from 10.0 mL to 13.5 mL, and multiple new bone lesions appeared, fulfilling DWIBS-PD criteria. Color scale as in Fig. 1

**Fig. 3:**
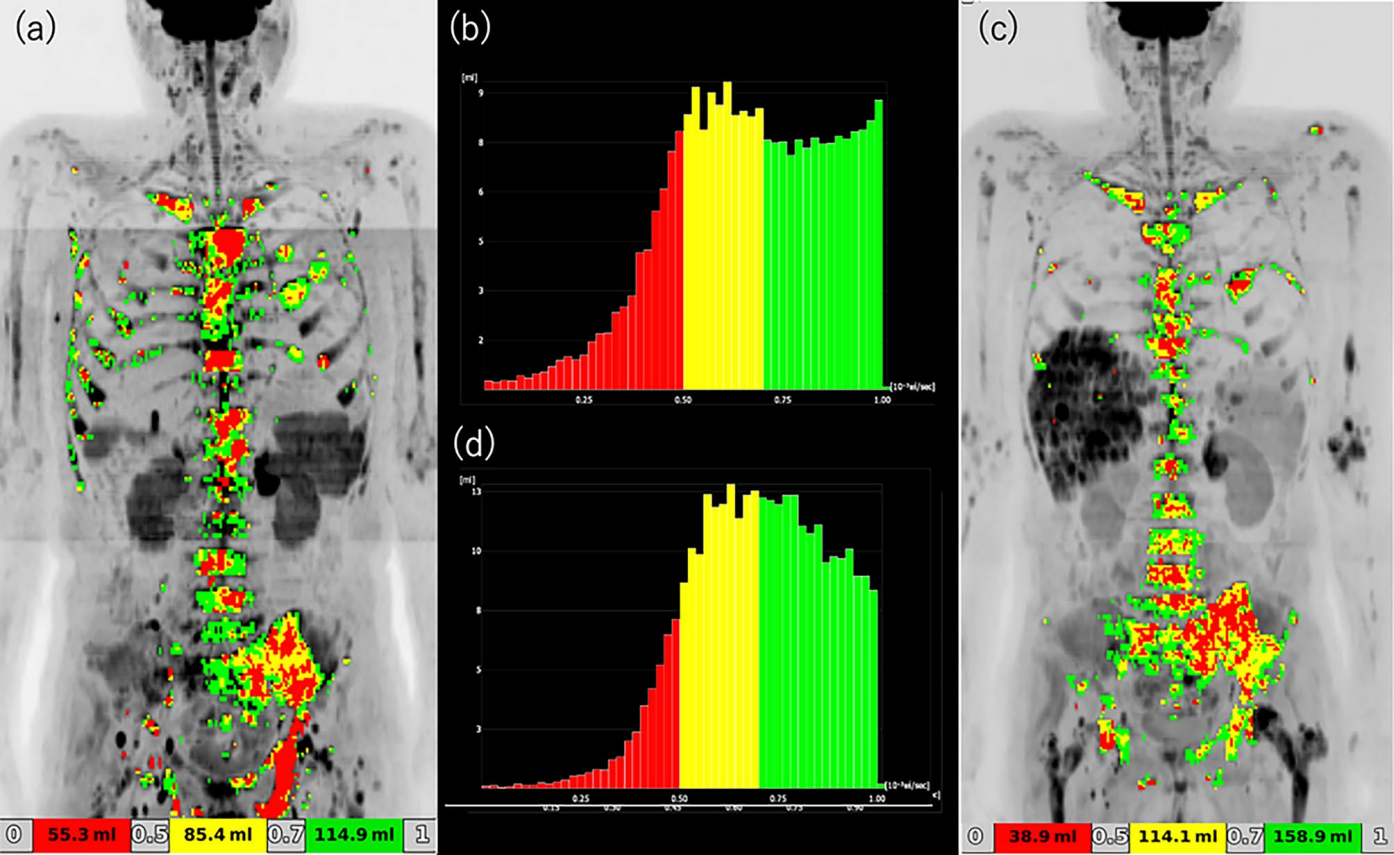
DWIBS-progressive disease (DWIBS-PD) case 2—visceral-metastasis progression. **a** Baseline DWIBS MIP with ADC colour overlay. **b** Baseline ADC histogram. **c** Follow-up DWIBS MIP after cycle 3 showing new visceral metastases (arrows). **d** Follow-up ADC histogram. Despite a modest reduction in low-ADC voxels, the tDV increased from 256 to 312 mL, and new liver metastases emerged, meeting the DWIBS-PD criteria. Color scale as in Fig. 1

**Fig. 4:**
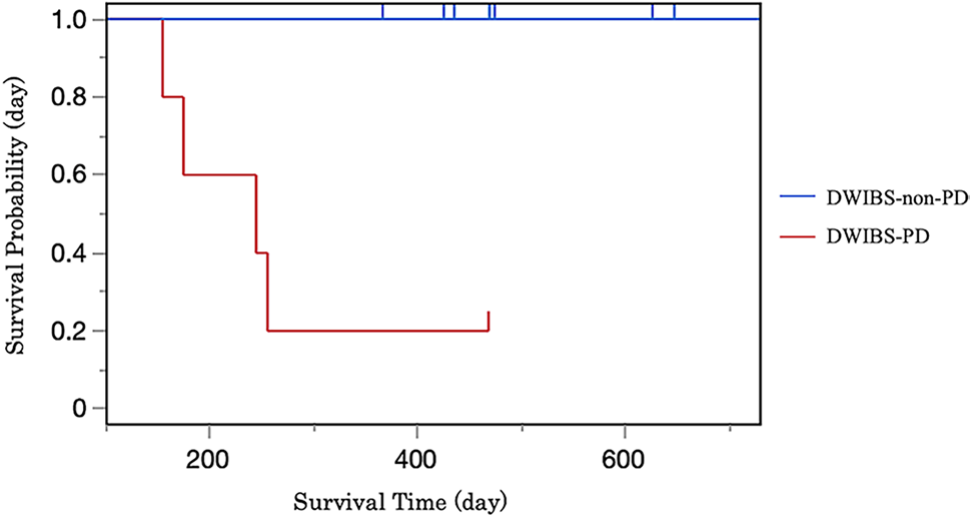
Kaplan–Meier survival curves. Kaplan–Meier survival analysis illustrating survival probability. The survival curve for the DWIBS-PD group showed a sharp decline within the first 300 days, whereas the DWIBS-non-PD group showed consistently higher survival probabilities throughout the observation period. One year survival rate was significantly lower in the DWIBS-PD group (*P* = 0.004), highlighting the prognostic value of DWIBS in evaluating the treatment response

Of the 15 patients, 5 (33%) were classified as DWIBS-PD, and the remaining 10 (67%) were collectively categorized as DWIBS-non-PD. Within this DWIBS-non-PD group, 3 achieved DWIBS-PR and 7 were judged to have DWIBS-SD. A typical DWIBS-PR case is shown in Fig. 1, while Figs. 2 and 3 illustrate representative DWIBS-PD cases. Of the 15 patients with CRPC and bone metastases, 11 survived for one year after treatment initiation, whereas 4 died within one year. All 4 of these patients belonged to the DWIBS-PD group, and 80% of the 5 DWIBS-PD patients died within one year, with a mean survival time of 174 days (Fig. 4). There were significant differences in PS score, DWIBS classification, and tDV increase volume between the two groups (Table 2). All the patients who died within 1 year had ECOG PS 2, whereas 82% of the 1-year survivors had PS 0. All the patients who died within 1 year were DWIBS-PD, whereas 10 of 11 patients (91%) who survived for more than one year were DWIBS-PR or DWIBS-SD. Patients with higher baseline tDV tended to have shorter survival. Representative ADC histograms for DWIBS-PR and DWIBS-PD cases are presented in Figs. 1, 2 and 3, respectively. In the PR case, the histogram demonstrated a noticeable reduction in red (< 0.5) and yellow (0.5–0.7) signal intensities after treatment. In contrast, the DWIBS-PD cases showed persistent dominance or even increase in low ADC components, with the emergence of new lesions or visceral metastases. These histogram patterns visually support the classification based on tDV.

**Table 2:** Key clinical characteristics stratified by 1-year survival

Variables	Overall	Survived < 1 year	Survived ≥ 1 year	P value
	*n *= 15	*n* = 4	*n* = 11	
Age (year), median (range)	72 (62–84)	70.5 (67–83)	76 (62–84)	0.600
ECOG performance status [0/1/2]	9/4/2	0/2/2	9/2/0	0.007
DWIBS RECIST response [DWIBS-PR/DWIBS-SD/DWIBS-PD]	3/7/5	0/0/4	3/7/1	0.004
tDV increase volume(mL), median (range)*	3.5 (−53.4–107.5)	54.45(5.4–107.5)	−7.2(−53.4–69)	0.019

*tDV* total diffusion volume, *PR* partial response, *SD* stable disease, *PD* progressive disease

^*^Change in total diffusion volume (tDV) from baseline to the 3-month follow-up DWIBS scan

Notably, in one DWIBS-PD case (Fig. 3), although low-ADC components slightly decreased, the tDV markedly increased and new visceral metastases emerged, meeting the criteria for progression. This highlights that tDV increase and new lesion appearance take precedence over histogram shift alone in DWIBS-based classification.

In addition to the parameters shown in Table 2, we also evaluated other clinical and laboratory parameters, such as BMI, Gleason score, pain status, bone scan index, PSA doubling time, baseline alkaline phosphatase (ALP), baseline PSA levels, and number of Ra-223 treatment cycles. None of these variables demonstrated a statistically significant relationship with 1-year survival (all *P* > 0.05). Detailed data for these additional factors are provided in Supplementary Table S1. Despite no significant differences, the 1-year survival group tended to have a longer PSA doubling time and a higher treatment completion rate than those who died within 1 year. Among DWIBS-PD, progression of bone lesions was observed in three patients and visceral metastasis in 2 patients.

### Clinical characteristics of DWIBS-PD

Patients in DWIBS-PD had a significantly lower performance score compared to those with DWIBS-non-PD group (Table 3). All the patients with ECOG PS 2 were classified as DWIBS-PD, while 50% of those with PS 1 and only 11% with PS 0 were DWIBS-PD. The DWIBS-non-PD group had a better performance status compared to DWIBS-PD group. There were no significant differences in pain, age, BMI, PSA doubling time, tDV before treatment, Gleason score, baseline ALP level, baseline PSA level, and BSI between DWIBS-PD group and DWIBS-non-PD group. Patients with high baseline PSA, ALP, and tDV values tended to have more advanced disease stage.

**Table 3:** Comparison of clinical characteristics

Variables	DWIBS-PD	DWIBS-non-PD	*P* value
	(*n* = 5)	(*n* = 10)	
Age (year), median (range)	72 (67–83)	74 (62–84)	0.959
ECOG performance status [0/1/2]	1/2/2	8/2/0	0.039
Pain [none/only during movement/constant]	2/1/2	8/1/1	0.279
BMI, median (range)	22.64 (16.4–29.1)	25.39 (19.3–30.3)	0.182
Gleason score [8/9/10/Unknown]	1/3/0/1	2/6/1/1	0.786
Pre-treatment ALP, median (range)	128 (14.1–833.5)	103 (53–347)	0.233
Pre-treatment PSA, median (range)	3.25 (1.30–254.2)	5.09 (0.44–58.5)	0.181
PSA Doubling Time (days), median (range)	59 (28–95)	96 (36–319)	0.178
Pre-treatment tDV (mL), median (range)	266.9 (14.1–833.5)	33.9 (2.3–694.2)	0.214
Pre-treatment Bone Scan Index (%)	1.13 (0–13.69)	0.595 (0–6.26)	0.264

*PD* progressive disease, *ALP* alkaline phosphatase, *PSA* prostate-specific antigen, *tDV* total diffusion volume

## Discussion

The effectiveness of Ra-223 therapy has primarily relied on bone scintigraphy and PSA kinetics, but these methods have limitations [12, 13]. Because Ra-223 therapy and bone scintigraphy tracers share a similar mechanism of accumulation in bone lesions, it is not possible to assess the effectiveness of Ra-223 therapy for bone metastases that appear negative on bone scintigraphy. Moreover, although bone scintigraphy has high sensitivity, it cannot evaluate visceral or soft tissue metastases. In contrast, DWIBS, which does not rely on bone metabolism, enables the simultaneous evaluation of bone, soft tissue, and organ lesions, offering a more holistic view of disease progression. The tDV, a quantitative measure of the total volume of bone metastases, serves as an even more objective indicator of prognosis than visual assessment. Furthermore, the incorporation of ADC histograms allowed for additional insight into the qualitative changes in diffusion profiles of bone lesions. Histograms showing reduction in lower ADC components were associated with favorable response, whereas persistent low-ADC dominance paralleled progression. This dual approach, volume-based and distribution-based, may enhance the interpretative value of DWIBS in therapeutic monitoring.

The rationale for using 1.0 × 10^−3^ mm^2^/s as the ADC threshold to define tDV is based on prior validation studies [14], which indicate that malignant bone lesions generally exhibit lower ADC values than benign lesions, with a cut-off of 920.5 × 10^−6^ mm^2^/s. In addition, the MET-RADS-P guidelines note that ADC values for bone metastases typically range from 700 × 10^−6^ mm^2^/s to 1400 × 10^−6^ mm^2^/s [15]. Guided by these findings, we determined that 1.0 × 10^−3^ mm^2^/s was the most appropriate threshold because it consistently matched the visually detected abnormal signals in automatically extracted regions on MIP images.

Although the threshold is still under debate, previous studies have confirmed that tDV is a reliable indicator demonstrating high intra- and interobserver reproducibility [16]. Furthermore, tDV is reported to be a valuable tool for evaluating treatment efficacy in prostate and breast cancer patients with bone metastases [16–18]. Although high incidence of false-positive findings with DWIBS has been reported in pediatric cancer patients, especially in bone marrow lesions caused by hyperplastic bone marrow signals during treatment response, the patients in this study were adults and did not undergo chemotherapy. Therefore, the results of this study demonstrated the utility and prognostic value of DWIBS in assessing both bone metastasis progression and treatment response in CRPC patients.

The average survival time of DWIBS-PD group was only 174 days, suggesting a limited effect of Ra-223 therapy. The most important predictor of poor response to Ra-223 therapy was ECOG PS, as all the patients with a PS 2 and half of those with a PS 1 were in the DWIBS-PD group. These results indicate that patients with disease progression at the midpoint of treatment are unlikely to benefit from Ra-223 therapy, and that treatment should be discontinued or switched to an alternative treatment. Furthermore, the DWIBS-PD group included patients whose bone lesions had not spread to any internal organs but were nonetheless resistant to Ra-223 therapy. Notably, an increase in tDV of 10% or more during treatment indicated that these patients’ prognosis was just as poor as that of patients with internal organ metastases. Thus, the mid-treatment evaluation of DWIBS provides important information to the clinicians for deciding treatment efficacy, in CRPC patients undergoing Ra-223 therapy.

### Limitation

First, to verify these results and improve the statistical accuracy, a larger multi-center study is needed. Nevertheless, this is the first study to evaluate the clinical usefulness of DWIBS in patients with CRPC undergoing Ra-223 therapy. As a post-hoc, exploratory analysis, we performed a multiple logistic regression using 4 variables (PS, DWIBS classification, tDV increase volume, and age) to further assess these factors. However, our small sample size (*n* = 15) led to quasi-complete separation and unstable parameter estimates, preventing us from obtaining reliable multivariable results. As such, the potential confounding effect of ECOG PS cannot be ruled out and should be evaluated with a larger number of cases in the future. Second, the DWIBS was assessed by a single radiologist, which may have introduced observer bias. Quantitative evaluation software helped to minimize observer bias, but reproducibility could be further improved by consensus among multiple radiologists. Third, therapeutic effect of Ra-223 was evaluated based on a single parameter. In recent years, the nomogram has been developed using multiple parameters to predict which patient groups are likely to respond to Ra-223 therapy [19]. By combining the nomogram, it would be possible to optimize the usefulness of DWIBS undergoing Ra-223 therapy.

## Conclusion

This study demonstrated that DWIBS is a valuable tool for predicting treatment response and prognosis in patients with CRPC undergoing Ra-223 therapy. This non-invasive imaging approach could support clinical decision-making and timely treatment modifications in patients undergoing Ra-223 therapy.

## Supplementary Information

Below is the link to the electronic supplementary material.

Supplementary file1 (DOCX 16 KB)

## References

[1] So A, Chin J, Fleshner N, Saad F. Management of skeletal-related events in patients with advanced prostate cancer and bone metastases: incorporating new agents into clinical practice. Canadian Urological Association journal = Journal de l’Association des urologues du Canada. Published online 2012. https://www.semanticscholar.org/paper/Management-of-skeletal-related-events-in-patients-So-Chin/d60ad76bfb9a750c683ee239b3b1fe5ab50b58d7. Accessed 19 Oct 2024. 10.5489/cuaj.12149 PMC3526633 23282666

[2] Vignani F, Bertaglia V, Buttigliero C, Tucci M, Scagliotti GV, Di Maio M. Skeletal metastases and impact of anticancer and bone-targeted agents in patients with castration-resistant prostate cancer. Cancer Treat Rev. 2016;44:61–73. 10.1016/j.ctrv.2016.02.002. 26907461 10.1016/j.ctrv.2016.02.002

[3] Brito AE, Etchebehere E. Radium-223 as an approved modality for treatment of bone metastases. Semin Nucl Med. 2020;50(2):177–92. 10.1053/j.semnuclmed.2019.11.005. 32172803 10.1053/j.semnuclmed.2019.11.005

[4] Vogelzang NJ, Parker C, Nilsson S, Coleman RE, O’Bryan-Tear CG, Shan M, et al. Updated analysis of radium-223 dichloride (Ra-223) impact on skeletal-related events (SRE) in patients with castration-resistant prostate cancer (CRPC) and bone metastases from the phase III randomized trial (ALSYMPCA). JCO. 2013;31(6_suppl):11–11. 10.1200/jco.2013.31.6_suppl.11.

[5] Van Der Zande K, Oyen WJG, Zwart W, Bergman AM. Radium-223 treatment of patients with metastatic castration resistant prostate cancer: biomarkers for stratification and response evaluation. Cancers. 2021;13(17):4346. 10.3390/cancers13174346. 34503156 10.3390/cancers13174346 PMC8431634

[6] Jee HB, Park MJ, Lee HS, Park MS, Kim MJ, Chung YE. Is Non-contrast CT adequate for the evaluation of hepatic metastasis in patients who cannot receive iodinated contrast media? PLoS ONE. 2015;10(7): e0134133. 10.1371/journal.pone.0134133. 26218533 10.1371/journal.pone.0134133 PMC4517761

[7] Kwee TC, Takahara T, Ochiai R, Nievelstein RAJ, Luijten PR. Diffusion-weighted whole-body imaging with background body signal suppression (DWIBS): features and potential applications in oncology. Eur Radiol. 2008;18(9):1937–52. 10.1007/s00330-008-0968-z. 18446344 10.1007/s00330-008-0968-z PMC2516183

[8] Takahara T, Imai Y, Yamashita T, Yasuda S, Nasu S, Cauteren MV. Diffusion weighted whole body imaging with background body signal suppression (DWIBS): technical improvement using free breathing, STIR and high resolution 3D display. Radiation medicine. Published online July 1, 2004. https://www.semanticscholar.org/paper/Diffusion-weighted-whole-body-imaging-with-body-and-Takahara-Imai/7772ce8c1fff127198a138e9dabf1276b7fb4d2c. Accessed 20 Oct 2024. 15468951

[9] Koh DM, Collins DJ. Diffusion-weighted MRI in the body: applications and challenges in oncology. Am J Roentgenol. 2007;188(6):1622–35. 10.2214/AJR.06.1403. 17515386 10.2214/AJR.06.1403

[10] Sartor O, Coleman RE, Nilsson S, Heinrich D, Helle SI, O’Sullivan JM, et al. An exploratory analysis of alkaline phosphatase, lactate dehydrogenase, and prostate-specific antigen dynamics in the phase 3 ALSYMPCA trial with radium-223. Ann Oncol. 2017;28(5):1090–7. 10.1093/annonc/mdx044. 28453701 10.1093/annonc/mdx044 PMC5406754

[11] Gallicchio R, Mastrangelo PA, Nardelli A, Mainenti PP, Colasurdo AP, Landriscina M, et al. Radium-223 for the treatment of bone metastases in castration-resistant prostate cancer: when and why. Tumori. 2019;105(5):367–77. 10.1177/0300891619851376. 31096849 10.1177/0300891619851376

[12] Nome R, Hernes E, Bogsrud TV, Bjøro T, Fosså SD. Changes in prostate-specific antigen, markers of bone metabolism and bone scans after treatment with radium-223. Scand J Urol. 2015;49(3):211–7. 10.3109/21681805.2014.982169. 25515952 10.3109/21681805.2014.982169

[13] Maruyama K, Utsunomia K, Nakamoto T, Kawakita S, Murota T, Tanigawa N. Utility of F-18 FDG PET/CT for detection of bone marrow metastases in prostate cancer patients treated with radium-223. Asia Ocean J Nucl Med Biol. 2018;6(1):61–7. 10.22038/aojnmb.2017.9896. 29333469 10.22038/aojnmb.2017.9896 PMC5765335

[14] Sun W, Li M, Gu Y, Sun Z, Qiu Z, Zhou Y. Diagnostic value of whole-body DWI with background body suppression plus calculation of apparent diffusion coefficient at 3 T versus 18F-FDG PET/CT for detection of bone metastases. Am J Roentgenol. 2020;214(2):446–54. 10.2214/AJR.19.21656. 31799866 10.2214/AJR.19.21656

[15] Parillo M, Mallio CA. The whole-body MRI reporting and data system guidelines for prostate cancer (MET-RADS-P), multiple myeloma (MY-RADS), and cancer screening (ONCO-RADS). Cancers. 2025;17(2):275. 10.3390/cancers17020275. 39858056 10.3390/cancers17020275 PMC11763526

[16] Blackledge MD, Tunariu N, Orton MR, Padhani AR, Collins DJ, Leach MO, et al. Inter- and intra-observer repeatability of quantitative whole-body, diffusion-weighted imaging (WBDWI) in metastatic bone disease. PLoS ONE. 2016;11(4):e0153840. 10.1371/journal.pone.0153840. 27123931 10.1371/journal.pone.0153840 PMC4849763

[17] Perez-Lopez R, Mateo J, Mossop H, Blackledge MD, Collins DJ, Rata M, et al. Diffusion-weighted imaging as a treatment response biomarker for evaluating bone metastases in prostate cancer: a pilot study. Radiology. 2017;283(1):168–77. 10.1148/radiol.2016160646. 27875103 10.1148/radiol.2016160646 PMC6140995

[18] Blackledge MD, Collins DJ, Tunariu N, Orton MR, Padhani AR, Leach MO, et al. Assessment of treatment response by total tumor volume and global apparent diffusion coefficient using diffusion-weighted MRI in patients with metastatic bone disease: a feasibility study. PLoS ONE. 2014;9(4):e91779. 10.1371/journal.pone.0091779. 24710083 10.1371/journal.pone.0091779 PMC3977851

[19] Kitajima K, Igeta M, Kuyama J, Kawahara T, Suga T, Otani T, et al. Novel nomogram developed for determining suitability of metastatic castration-resistant prostate cancer patients to receive maximum benefit from radium-223 dichloride treatment-Japanese Ra-223 therapy in prostate cancer using bone scan index (J-RAP-BSI) trial. Eur J Nucl Med Mol Imag. 2023;50(5):1487–98. 10.1007/s00259-022-06082-3. 10.1007/s00259-022-06082-3 36539508

