# Supplementary material for: Prognostic value of whole-body diffusion-weighted imaging with background body signal suppression in CRPC patients undergoing Ra-223 therapy: an exploratory analysis
Source: Jpn J Radiol. 2025 Jun 17;43(10):1688–95. doi: 10.1007/s11604-025-01817-2 (PMC12479555; doi:10.1007/s11604-025-01817-2)
Supplement: Supplementary file 1 — Supplementary file1 (DOCX 16 KB) [file 11604_2025_1817_MOESM1_ESM.docx]

Supplementary Table S1. Additional Clinical Variables by 1-Year Survival

|  | Overall | Survived < 1 year | Survived ≥ 1 year | P value |
| --- | --- | --- | --- | --- |
| Variables | n=15 | n=4 | n=11 |  |
| Age (year), median (range) | 72 ( 62-84 ) | 70.5 ( 67-83 ) | 76 ( 62-84 ) | 0.600 |
| Pain [None/Only during movement/Constant] | 10 / 2 / 3 | 2 / 1 / 1 | 8 / 1 / 2 | 0.653 |
| BMI (kg/m^2^), median (range) | 24.09(16.4 - 30.3) | 20.54(16.4-29.1) | 24.5(19.3 - 30.3) | 0.296 |
| Number of treatments [6/<6] | 9 / 6 | 1 / 3 | 8 / 3 | 0.175 |
| Gleason score [8/9/10/Unknown] | 3 / 9 / 1 / 2 | 1 / 3 / 0 / 0 | 2(18.2%)/ 6(54.5%/ 1(9.1%) | 0.786 |
| Pre-treatment ALP (IU/L), median (range) | 108(53 - 618) | 204.4(93 - 618) | 108(53 - 331.4) | 0.330 |
| Pre-treatment PSA (ng/mL), median (range) | 4.59 ( 0.44 - 254.2 ) | 3.02(1.30 - 254.2) | 5.59(0.44 - 164.5) | 0.695 |
| PSA Doubling Time (days), median (range) | 89.6 ( 28 - 319.2 ) | 56(28-95.2) | 95.2(36.4 - 319.2) | 0.170 |
| Pre-treatment tDV (mL), median (range) | 180 ( 2.3-833.5 ) | 261.3(14.1-411.9) | 35.1(2.3 - 833.5) | 0.695 |
| Pre-treatment Bone Scan Index (%) | 0.63 ( 0 - 13.69 ) | 2.38(0 - 13.69) | 0.63(0 - 6.26) | 0.647 |
| BMI: body mass index, ALP: Alkaline Phosphatase, PSA: Prostate-Specific Antigen | | | | |
